# *mcr-1* identified in Avian Pathogenic *Escherichia coli* (APEC)

**DOI:** 10.1371/journal.pone.0172997

**Published:** 2017-03-06

**Authors:** Nicolle Lima Barbieri, Daniel W. Nielsen, Yvonne Wannemuehler, Tia Cavender, Ashraf Hussein, Shi-gan Yan, Lisa K. Nolan, Catherine M. Logue

**Affiliations:** 1 Department of Veterinary Microbiology and Preventive Medicine, College of Veterinary Medicine, Iowa State University, 1802 University Blvd; VMRI #5 Ames, IA, United States of America; 2 Department of Avian and Rabbit Medicine, Faculty of Veterinary Medicine, Zagazig University, Zagazig, Egypt; 3 School of Bioengineering, Qilu University of Technology, Changqing District, Jinan, Shandong Province, P. R. China; Cornell University, UNITED STATES

## Abstract

Antimicrobial resistance associated with colistin has emerged as a significant concern worldwide threatening the use of one of the most important antimicrobials for treating human disease.

Here, we examined a collection (n = 980) of Avian Pathogenic *Escherichia coli* (APEC) isolated from poultry with colibacillosis from the US and internationally for the presence of *mcr-1* and *mcr-2*, genes known to encode colistin resistance. Included in the analysis was an additional set of avian fecal *E*. *coli* (AFEC) (n = 220) isolates from healthy birds for comparative analysis. The *mcr-1* gene was detected in a total of 12 isolates recovered from diseased production birds from China and Egypt. No *mcr* genes were detected in the healthy fecal isolates. The full *mcr-1* gene from positive isolates was sequenced using specifically designed primers and were compared with sequences currently described in NCBI. *mcr-1* positive isolates were also assessed for phenotypic colistin resistance and extended spectrum beta lactam phenotypes and genotypes. This study has identified *mcr-1* in APEC isolates dating back to at least 2010 and suggests that animal husbandry practices could result in a potential source of resistance to the human food chain in countries where application of colistin in animal health is practiced.

## Introduction

The emergence of *mcr-1* and *mcr-2* genes associated with colistin resistance in *Enterobacteriaceae* has gained international attention in light of its potential as a human health threat because of the ability of these organisms to resist one of mankind’s last drugs of resort—colistin. Reports from the USA have identified *mcr-1* in human isolates of *E*. *coli* from a patient with a urinary tract infection [1] and another that was also associated with a clinical case [2]; in addition isolates have also been found associated with swine [3, 4]. Of greater significance is that in the human case, the patient reported no history of travel in the previous five months, while the detection of *mcr-1* in swine would suggest that *mcr-1* may already be present in production animals in the US with the potential for this resistance to enter the human food chain.

An explosion of reports has emerged in light of the first report of the detection of *mcr-1* associated resistance in isolates of *E*. *coli* from animals and humans in China [5]. Recently, researchers have rushed to assess historical isolates in an effort to identify potential emergence dates for *mcr* and current reports have identified isolates harboring *mcr-1* as far back as 1980 [6]. Worldwide reports have identified of *mcr-1* in a range of *Enterobacteriaceae* from human and animal hosts including *Escherichia coli*, *Salmonella*, *Klebsiella* and other Gram negative organisms [7–14]. Researchers have identified the genomic locations of *mcr-1* which include chromosomal integration [15], while others report that *mcr-1* is mobile, being frequently linked with a range of plasmid types including Inc I2, Inc P, Inc FIP, Inc F and Inc HI2 as well as some Inc X4 types [1, 3, 5, 14–18]. Perhaps the biggest concern with regards to the rapid recognition of the emergence of *mcr-1* is the association between *mcr* and other resistance elements such as extended spectrum beta-lactam (ESBL) antimicrobial agents [1, 8, 19, 20], the carbapenemases [21, 22] and heavy metals such as copper [23] and more recently linked with New Delhi Metallo β-Lactamase (NDM) [24].

*mcr-1*-associated resistance in *E*. *coli* linked with both healthy and diseased production animals and wildlife have been documented worldwide [5, 12, 25–28], but reports of the association between *mcr-1* and colibacillosis associated disease in poultry currently appears to be limited. Assessment of *mcr-1* associated resistance in APEC is warranted to determine potential sources of *mcr-1* to the human food chain but also to determine the potential risk for treatment of poultry disease, putting one of the world’s most important and cheapest sources of protein at risk.

One study from South Africa identified *mcr-1* in APEC [29] and a second from China [30] identified two *E*. *coli* isolates harboring *mcr-1* resistance in a Muscovy duck, indeed a recent genome from Germany identified *mcr-1* in a 2010 strain of avian ExPEC responsible for septicemia in a broiler chicken [31]. Of significant concern is the purported link between APEC-contaminated retail poultry meat, human UTIs and other diseases [32–35], which suggest that poultry harboring colistin-resistant APEC could be a potential food-borne vehicle of *mcr* genes for human disease.

We are currently assessing an Avian Pathogenic *E*. *coli* (APEC) collection in association with collaborators around the world for traits associated with pathogenicity and antimicrobial resistance. In light of the recent reports of the emergence of *mcr*, we rapidly screened our historical collections for *mcr-1*. Here, we report the screening of 675 APEC isolated from production birds in the US dating back over a 20 year period [36]; also we have included 29 isolates from Central America (Mexico), 87 isolates from South America (Brazil 47; Peru 12; Venezuela 10; Colombia 18) [37–39], 23 isolates from the Caribbean (Dominican Republic 23); 30 isolates from Africa (Egypt) [40], 31 isolates from China; 72 isolates from Turkey and 30 isolates from Italy. All APEC isolates were recovered from the lesions of production birds (including broilers, layers, breeder flocks, ducks and geese) showing signs of colibacillosis ranging from perihepatitis, pericarditis, airsacculitis, cellulitis, omphailitis, colisepticemia, swollen head syndrome and other such colibacillosis traits [41]. All isolates had been previously confirmed as APEC using the pentaplex PCR reaction [42]. Included in the analysis were an additional 220 isolates from the feces of healthy broilers and turkeys recovered in the US and Egypt these isolates were included for comparative purposes. All isolates examined were subjected to phylogenetic typing [43], and most were serogrouped.

## Materials and methods

### Isolate collection

This study was an observational screen of a collection of APEC and AFEC collected from around the world and consisted of APEC strains recovered from the lesions of production poultry diagnosed with colibacillosis (APEC) and isolates recovered from the feces of healthy production birds (AFEC). Table 1 shows the source of the strains and years of isolation and the production bird types associated with the collection used in the analysis.

**Table 1:** Source of isolates examined for the *mcr-1* and *mcr-2* genes used in this study.

Continent	Isolate Source	# Tested for *mcr*	Date	Bird Types
N. America	USA—APEC	452	1980’s to 2005	broilers, layers, turkeys
	USA–APEC–D lab	223	2005–2011	avian
Central America	Mexico–APEC	29	2010–2012	broilers
Caribbean	Dominican Republic—APEC	23	2011	broilers
S. America	Brazil—APEC	47	2006–2015	broilers
	Venezuela–APEC	10	2011	broilers
	Peru–APEC	12	2011	broilers
	Colombia—APEC	18	2011	broilers, layers
Europe	Italy–APEC	33	2004–2007	broilers
	Turkey—APEC	72	2014–2015	broilers
Africa	Egypt–APEC	30	2010	broilers
Asia	China–APEC	31	2012–2015	broilers, duck, geese
N. America	USA—AFEC	200	Before 2005 and 2010	broilers, turkeys
Africa	Egypt—AFEC	20	2010	broilers
	**Total**	**1200**		

### PCR analysis

All isolates were screened for the presence of the *mcr-1* using the protocol recently reported by Liu et al [5] and the following primers designed to target the *mcr-1* gene CLR5-F (5ʹ-CGGTCAGTCCGTTTGTTC-3ʹ) and CLR5-R (5ʹ-CTTGGTCGGTCTGTA GGG-3’) as described by Cavaco and Hendricksen (http://www.crl-ar.eu/data/images/protocols/*mcr-1*_pcr_protocol_v1_dec2015.pdf). A 25ul PCR reaction was carried out with the following amplification conditions 94°C 15 min; 25 cycles of 94°C 30 sec; 58°C 90 sec; 72°C 60 sec with a final extension of 72°C for 10 min. PCR generated amplicons were run on a 2% agarose gel at 100 v for 2h and stained in ethidium bromide for visualization of the 309 bp product. PCR products positive for the *mcr-1* gene were treated with ExoSAP-IT® (Affymetrix, Santa Clara, CA) and submitted to ISU’s DNA facility for Sanger sequencing.

A second set of primers designed to identify the *mcr-2* gene [44] were used to screen the collection for the presence of the novel *mcr-2* variant. The primer sequences used were: MCR2-IF 5’ TGTTGCTTGTGCCGATTGGA 3’ and MCR2-IR 5’ AGATGGTATTGTTGGTTGCTG 3’, with cycling conditions and gel electrophoresis as described above. PCR products generated of 567 bp were considered potentially positive for *mcr-2* and subjected to sequencing as described above.

A new set of primers were designed to amplify the complete *mcr-1* and *mcr-2* genes simultaneously by mining gene sequences already available in NCBI (KU88614 and NG_051171.1) (see http://www.ncbi.nlm.nih.gov/nuccore/ku886144 and [44]) and using Primer3 software. The same strains that were positive for the *mcr-1* fragment were amplified for the full (1311 bp) gene using the new universal primers: *mcr1-2* universal F ACTTATGGCACGGTCTATGATAC and *mcr1-2* universal R CCGCGGTGACATCAAACA. All PCR amplifications were carried out under the following conditions 94°C for 10 minutes followed by 30 cycles of 94°C for 30S; 58°C for 30S and 72°C for 2 min; with a final extension of 72°C for 10 minutes. PCR products were run on an agarose gel as described above.

We were unable to use these primers to detect *mcr-2* variants as none were detected in our study, but these primers may be useful for the simultaneous screen of *mcr-1* and *mcr-2*, and the fragment generated is large enough at 1311 base pairs (approximately 80.6% and 72.2% of the *mcr-1* and *mcr-2* genes respectively) to allow comparison.

The full gene PCR product was cleaned using ExoSAP-IT® and submitted to ISU’s DNA facility for Sanger sequencing of both strands. Sequences generated were imported into Geneous® software and aligned to compare across the isolates positive for the fragment.

### NARMS analysis

Antimicrobial resistance analysis of the isolates positive for the *mcr* gene was carried out using the broth microdilution assay using the National Antimicrobial Resistance Monitoring System (NARMS) [45] and minimum inhibitory concentrations (MICs) recorded for each strain based on growth/ no growth in the wells of the plate. All MICs recorded were compared against the accepted breakpoints for *E*. *coli* recovered from animals using the CLSI and NARMS criteria (see http://www.ars.usda.gov/Main/docs.htm?docid=6750&page=3). Antimicrobial resistance/ susceptibility was examined for the following antimicrobials: Amikacin (AMI); Ampicillin (AMP); Azithromycin(AZI); Amoxicillin/Clavulanic acid (AUG); Ceftriaxone (AXO); Chloramphenicol (CHL); Ciprofloxacin (CIP); Trimethoprim/Sulfamethoxazole (SXT); Cefoxitin (FOX); Gentamicin (GEN); Kanamycin (KAN); Nalidixic acid (NAL); Sulfisoxazole (FIS); Streptomycin (STR); Tetracycline (TET); Ceftiofur (TIO).

### Extended spectrum beta lactam (ESBL) resistance screening

Isolates positive for ceftriaxone and ceftiofur resistance were screened for ESBL associated resistance genes using PCR protocols and primers described for *bla*_*TEM*_, *bla*_*SHV*_, *bla*_*CMY*_, *bla*_*CTX-M*_ and *bla*_*OXA*_ [46–49].

### Antimicrobial susceptibility analysis

In an effort to assess the role of colistin resistance in strains positive for the *mcr* gene all strains were subjected to antimicrobial susceptibility analysis to colistin sulfate (Alfa Aesar, Ward Hill, MA) using broth microdilution and agar dilution assays. Overnight cultures of each strain were grown on Tryptone Soya Agar (TSA) plates and colonies selected and adjusted to a OD 0.5 McFarland in sterile water using an nephelometer (Sensititre); then 10ul of the suspension was added to 11 mls of Mueller Hinton (MH) broth which was used to inoculate the broth microdilution plates and the agar dilution plates.

The broth microdlution and agar dilution plates tested antimicrobial resistance to colistin at the following dilution range 0.5 to 32 ug/ml. Once all plates were inoculated as appropriate they were incubated at 37°C for 18h. Plates were observed for growth and minimum inhibitory concentrations (MIC’s) were defined as the lowest concentration of antimicrobial to inhibit growth of the test strains.

### Plasmid replicon detection

Plasmid screening of all isolates was assessed using the plasmid replicon typing protocols described by Carattoli et al. and Johnson et al. [50, 51] with the inclusion of replicons associated with newly defined X replicon types 1–4 [52] and X5 [53] and IncI2 [54].

### Plasmid extraction

To determine the location of the *mcr-1* gene, plasmids were extracted from all strains using the MinElute gel extraction kits (Qiagen, CA) with minor modifications, after resuspension of the pellet with water (step 7) instead of passing the plasmid suspension through the column provided in the kit, a QIAquick PCR purification kit as recommended by the supplier was used. Plasmid DNA was run on a pulse field gel electrophoresis (PFGE) gel in order to resolve the plasmids using protocols described by Tivendale et al 2004 [55].

### Plasmid conjugation studies

Plasmid conjugation assays were carried out using two separate approaches to determine the location of the *mcr-1* gene.

#### Conjugation

Isolates were grown overnight in Brain Heart Infusion (BHI) broth at 37˚C. Conjugation was then facilitated by mixing the donor culture with a culture of a recipient strain, *E*. *coli* 1932 (a nalidixic resistant, lactose negative strain devoid of plasmids with the capability to accept plasmids). Mixed cultures (1:1 ratio) were incubated at 37˚C for 1 and 24 hours before they were plated out on selective MacConkey agar (MAC) containing 4 ug/ml of colistin. Plates were incubated at 37˚C for 18–24 hours and transconjugants were selected as lactose negative colonies.

#### Electroporation

*E*. *coli* 1932 electro-competent cells were electroporated with 2 uL of purified plasmid (described above). They were allowed to recover with incubation at 37˚C with shaking for 90 minutes followed by plating out on Luria Bertani (LB) agar supplemented with 4 ug/ml of colistin. Plates were incubated at 37˚C for 18–24 hours and transformants were selected.

All transconjugants/transformants generated by both approaches were screened for the presence of *mcr-1* using PCR as described above.

## Results

The *mcr-1* gene was detected in 12 isolates from 980 isolates of APEC examined in this study (1.22% prevalence). Eight of the 12 isolates positive for the *mcr-1* gene were recovered from poultry (chicken) diagnosed with colibacillosis in China and the remaining four isolates were recovered from chickens diagnosed with colibacillosis in Egypt. No isolates from the USA or other continents and countries examined showed any *mcr-1* genes. None of the AFEC strains from the US or Egypt possessed *mcr-1*. Screening for the *mcr-2* gene also failed to detect this gene in any of the collection screened (APEC or AFEC).

Sequence analysis of the small gene fragment (309 bp) and then the larger gene fragment (1311 bp) showed 100% identity with *mcr-1* gene sequences currently available in NCBI. S1 Fig shows the nucleotide alignment of all 12 strains for the whole gene fragment when aligned using Geneous®. S2 Fig shows the protein alignment of the same twelve strains which also show 100% identity match with *mcr-1* available in NCBI (accession NG_051171.1).

Table 2 shows the characteristics of the 12 isolates that were found to be positive for the *mcr-1* gene. Isolates dating back as far as 2010 from Egypt were positive for the gene. Phylogenetic types identified among the 12 isolates varied, with strains being identified as phylogenetic types A, B1 and F. Serogroups and replicon types detected were also variable; however, replicon type FIB was detected in 11 of the 12 isolates; replicon type IncI2 in 10 of the 12 and replicon type I1 was common in eight. None of the isolates were positive for any of the X replicon types (X1-X5).

**Table 2:** Characteristics of *mcr-1* containing APEC.

ID	Source	Year	County of Origin	Syndrome	Phylotype	O Type	Replicon Type
7-49-1	chicken	2010	Egypt	colisepticemia	A	O157	FIB; L/M; IncI2
7-49-2	chicken	2010	Egypt	colisepticemia	F	O158	P; FIB; I1; IncI2
7-49-5	chicken	2010	Egypt	colisepticemia	B1	M	FIB; I1
7-49-8	chicken	2010	Egypt	colisepticemia	F	neg	FIA; FIB; I1; IncI2
8-5-27	chicken	2014	China	pericarditis, parahepatitis, airsacculitis	F	O11	FIB; I1; IncI2
8-5-31	chicken	2014	China	pericarditis, parahepatitis, airsacculitis	A	O6	I1; N; IncI2
8-6-1	chicken	2014	China	pericarditis, parahepatitis, airsacculitis	B1	O15	A/C; FIB; IncI2
8-6-3	chicken	2012	China	pericarditis, parahepatitis, airsacculitis	B1	O8	FIB; I1; N; IncI2
8-6-4	chicken	2012	China	pericarditis, parahepatitis, airsacculitis	B1	O11	FIB; I1; N; IncI2
8-6-5	chicken	2012	China	pericarditis, parahepatitis, airsacculitis	B1	neg	B/O; FIB; N
8-6-9	chicken	2012	China	pericarditis, parahepatitis, airsacculitis	B1	O111	FIB; I1; IncI2
8-6-12	chicken	2013	China	pericarditis, parahepatitis, airsacculitis	A	O6	B/O; FIB; N; IncI2

neg–isolate did not react with standard antisera or PCR amplification.

M–mixed reaction to different antisera.

Table 3 shows the NARMS results for all 12 isolates found to be positive for the *mcr-1* gene. All isolates of interest showed high levels of multi-drug and multi-class resistance. In addition, all twelve isolates showed high ceftriaxone and ceftiofur resistance, which are used as potential indicators of beta lactam and extended spectrum beta lactam (ESBL) resistance. Confirmation of resistance was corroborated by carriage of genes related to beta lactams and ESBLs including the *bla*_*TEM*_, *bla*_*SHV*_, *bla*_*CMY*_, *bla*_*CTX-M*_ *and bla*_*OXA*_ genes (see Table 4).

**Table 3:** NARMS analysis of isolates positive for the *mcr-1* gene.

	> = 32	> = 16	> = 64	> = 64	> = 32/16	> = 8	> = 32	> = 4	> = 512	> = 4/76	> = 32	> = 32	> = 1	> = 32	> = 16	> = 64
Isolate	AZI	GEN	KAN	STR	AUG2	XNL	FOX	AXO	FIS	SXT	AMP	CHL	CIP	NAL	TET	AMI
7-49-1	-	16	>64	>64	8	>8	8	>64	>256	>4	>32	8	>4	>32	>32	1
7-49-2	-	>16	>64	>64	16	>8	8	>64	>256	>4	>32	16	>4	>32	>32	1
7-49-5	-	>16	< = 8	64	8	>8	8	64	>256	0.5	>32	>32	>4	>32	>32	1
7-49-8	-	1	>64	>64	8	>8	4	>64	>256	>4	>32	4	>4	>32	32	4
8-5-27	8	16	-	>64	8	>8	8	64	>256	>4	>32	>32	>4	>32	>32	-
8-5-31	8	1	-	>64	8	>8	8	>64	>256	>4	>32	>32	>4	>32	>32	-
8-6-1	4	>16	-	>64	>32	8	>32	64	>256	>4	>32	>32	>4	>32	>32	-
8-6-3	4	1	-	>64	8	>8	16	64	>256	>4	>32	>32	>4	>32	>32	-
8-6-4	4	1	-	>64	8	>8	16	64	>256	>4	>32	>32	>4	>32	>32	-
8-6-5	16	>16	-	>64	8	>8	16	64	>256	>4	>32	>32	>4	>32	>32	-
8-6-9	16	>16	-	>64	8	>8	>8	>64	>256	>4	>32	>32	>4	>32	>32	-
8-6-12	16	>16	-	>64	8	>8	4	>64	>256	>4	>32	>32	>4	>32	>32	-

AZI–azithromycin; GEN–gentamicin; KAN–kanamycin; STR–streptomycin; AUG2 –amoxicillin/clavulanic acid; XNL- ceftiofur; FOX–cefoxitin; AXO–ceftriaxone; FIS–sulfisoxazole; SXT–trimethoprim sulfamethoxazole; AMP–ampicillin; CHL–chloramphenicol; CIP–ciprofloxacin; NAL–nalidixic acid; TET–tetracycline; AMI–amikacin;— = not tested for this drug as not available on the panel used. Upper line of table indicates breakpoints used to define resistance except for AZI where the CDC criteria were used. All criteria are adopted from the Clinical Laboratory Sciences Institute (CLSI) M100 document.

**Table 4:** Beta lactam and ESBL-associated antimicrobial resistance genes detected in strains positive for *mcr-1*.

Strain ID	*bla*_*TEM*_	*bla*_*SHV*_	*bla*_*CMY*_	*bla*_*CTX-M*_	*bla*_*OXA*_
7-49-1	+	-	-	+	-
7-49-2	+	-	-	+	-
7-49-5	+	-	-	+	-
7-49-8	+	-	-	+	-
8-5-27	-	-	-	-	-
8-5-31	-	-	-	+	-
8-6-1	-	-	+	-	+
8-6-3	-	-	-	+	+
8-6-4	-	-	-	+	+
8-6-5	+	-	-	+	+
8-6-9	+	-	-	-	+
8-6-12	+	-	-	+	+

Table 5 shows the colistin resistance profiles of *mcr-1* positive isolates when assayed using broth microdilution and agar dilution assays. Overall, both methods were relatively concordant and where there was disagreement, it was no more than one dilution difference. All isolates were considered resistant to colistin (at least 2–8 fold greater resistance), when a break point of > 2 ug/ml was used as recommended by EUCAST (http://www.eucast.org/clinical_breakpoints/).

**Table 5:** Colistin resistance among *mcr-1* positive isolates of APEC.

Isolate	Broth microdilution ug/ml	Agar dilution ug/ml
7-49-1	8	16
7-49-2	8	8
7-49-5	8	8
7-49-8	8	16
8-5-27	4	8
8-5-31	16	16
8-6-1	8	8
8-6-3	8	8
8-6-4	8	8
8-6-5	4	8
8-6-9	4	8
8-6-12	8	16

A breakpoint of >2 ug/ml was considered resistant following EUCAST guidelines http://www.eucast.org/clinical_breakpoints/

## Discussion

This study was carried out to assess the prevalence of colistin-associated resistance linked with the *mcr-1* and *mcr-2* genes in APEC from poultry from different world regions. Much speculation and attention has been paid to the detection of *mcr-1* genes and colistin resistance in *Enterobacteriace* because of concerns for the emergence of resistance to one of the last drugs of resort for treatment of disease in humans [56]. Speculation as to the source of colistin resistance and when it emerged has resulted in a deluge of research and retrospective papers searching for *mcr* and assessing dates of emergence. The entire APEC examined in our collection came from production birds diagnosed with colibacillosis with some of our US isolates dating back over 30 years. More recent isolates from South America, Mexico, Europe, Egypt and China were collected within the last 5 years.

Overall, *mcr-1* was detected in 12 isolates of APEC from a collection of 1200 APEC and AFEC isolates examined (1% prevalence). This level is relatively low but reflects a prevalence of about 25% among APEC isolates from China tested and about 13% from Egypt (amongst APEC). These numbers are, however, a poor reflection of overall prevalence of *mcr-1* in these countries as they are based on an analysis of 30 APEC isolates and may not be reflective of the true nature of *mcr-1* prevalence in these countries. Regardless, detection of *mcr-1* in disease associated *E*. *coli* of production birds is, however, concerning as the presence of *mcr-1* poses two threats: the health and welfare of the birds from the point of view that they may be harder to treat should an outbreak of infection occur and from the potential risk of consumer exposure from production birds that enter the food chain.

All twelve isolates examined in this study were of either phylogenetic type A, B1 or F. Phylogenetic types B2 and D were considered virulent by Clermont and colleagues [57], while later work by Clermont et al [43] recognized new phylogenetic types including groups C, E and F. Based on our data in this study, it would appear that for APEC at least, phylogenetic types A, B1 and F may also have pathogenic potential. Also of interest, a range of serogroups were detected among the *mcr-1* positive strains, reflecting the diversity of APEC as has been reported by others [36]. Similarly, plasmid replicon typing found that most isolates possessed an FIB type, IncI2 or I1 or all three (7 of 12), while FIB was detected in 11 of 12 strains. Plasmid types linked with carriage of *mcr-1* previously have included Inc I2, Inc P, Inc FIP, Inc F and Inc HI2 and some Inc X4 types [1, 5, 14–18, 54].

The detection of *mcr-1* associated colistin resistance in production animals is not new and multiple reports have linked *mcr-1* with *Enterobacteriaceae* of production animals including veal, swine, and poultry [5, 12, 25–27], which would suggest that colistin-resistant *Enteroabcteriace* have already become established in livestock and may pose a potential threat to consumers through the consumption of contaminated meat and other products. The presence of colistin-resistant organisms also reflects the application of colistin in some countries as an antimicrobial agent in feed. Reports from China by Liu and colleagues [5] noted that China is one of the largest users of colistin in animal agriculture, which may account for the first recognition of emergence of *mcr* in China, and Shen et al [6] noted that emergence of *mcr* in animal production may have occurred as a far back as the 1980s when colistin was first used in China. More recent reports from China by Wang and colleagues indicate that from April of 2017 use of colistin as a growth promoter there will cease [24].

It would appear that colistin is also available for use in animal agriculture in Egypt and has application in poultry (including treatment of colibacillosis), calves and rabbits (http://egypt.msd-animal-health.com/products/124_120825/productdetails_124_121485.aspx). Previous reports of *mcr-1* associated resistance in Egypt found *mcr-1* in an isolate of *E*. *coli* from a cow displaying subclinical mastitis[58] and in a human clinical case associated with bacteremia [59] suggesting that *mcr* associated resistance would also appear to be emergent in Egypt where the isolates of the present study were sourced.

This study was also able to review the *mcr-2* prevalence among our collection and we were unable to detect the gene in any isolates examined. Using the published gene sequences of *mcr-1* and *mcr-2* we identified significant overlap in their sequences that allowed development of a universal set of primers to rapidly screen for both genes and allow for comparative analysis of the 1311 bp fragment. This fragment covers nucleotides 327–1550 of KU886144’s 1626 nucleotides. Although our collection did not detect any *mcr-2* genes we wish to share these universal primers with the community to assess their usefulness.

The 12 strains positive for *mcr-1* were found to be resistant to a considerable number of antimicrobial agents using NARMS. Most were resistant 10 or greater antimicrobials. Of these strains three were resistant to 6 classes of drugs, 8 strains were resistant to 7 classes and 1 isolate was resistant to 8 classes. High levels of resistance to multiple types and classes of antimicrobials appears to be common with *mcr-1* containing strains and may be reflective of practice or issues in treating multidrug resistant strains that are causing disease in flocks in the first place. Regardless, the high levels of resistance observed and the types of classes of resistance observed is concerning. Also of concern is the potential for multi-drug-resistant strains to donate resistance to commensals or other organisms present in the environment or to allow their selection through application of agents to treat or control disease. Lastly, these strains were isolated from food producing birds which raises concerns for entry to the food chain, where there is risk of consumer exposure.

In association with the NARMS analysis, we investigated the relationship between *mcr-1* possessing strains and their potential for beta lactam and Extended Spectrum Beta Lactam (ESBL) resistance. NARMS analysis identified potential ESBL-associated resistance based on phenotypic resistance to ceftriaxone and ceftiofur and this was confirmed genotypically by possession of the beta lactam and ESBL associated genes with *bla*_*TEM*_, *bla*_*CTX-M*_ or *bla*_*OXA*_ being the most common. Most isolates appeared to carry two *bla* genes simultaneously with *bla*_*TEM*_ and *bla*_*CTX-M*_, being common while at least two strains carried three *bla* genes. Other researchers have highlighted the ESBL resistance as a concern especially in human strains [1]. Similarly, ESBL resistance associated with *mcr-1* has been observed in calves and poultry [8, 26] and in *Enterobacteriaceae* harboring plasmids bearing ESBL associated resistance genes [16, 19, 20]. Some workers have suggested that *mcr-1* and ESBL may be co-selected, sharing the same plasmid hosts [16, 19, 20]. All of the strains examined possessed ESBL-associated resistance, with 9 of 12 strains harboring *bla*_*CTX-M*_. Grami and colleagues [26] demonstrated that *E*. *coli* of healthy chickens bore plasmids harboring *bla*_*CTX-M*_ but these same plasmids also harbored *mcr-1* and were multi-resistant to phenicols, tetracycline, sulfonamides, and quinolones. In a similar study in Denmark, *E*. *coli* recovered from chicken meat that harbored *mcr-1* also harbored plasmids bearing *bla* genes such as *bla*_*TEM*_, *bla*_*CMY*_ and *bla*_*SHV*_ [25] confirming that these resistance traits appear to travel together.

Analysis of colistin resistance using the broth microdilution assay or the agar dilution assay correlated well and indicated most of the isolates had MICs >2 ug/ml when the EUCAST criteria were applied, all isolates examined in this study possessing the *mcr-1* gene had MICs significantly greater than the recognized breakpoint.

Despite our best efforts, at this time we have been unable to determine the genomic location of *mcr-1* among the colisitn-resistant APEC of our collection. Conjugation assays have failed to transfer resistance to a recipient strain and have only been successful by electroporation suggesting that the *mcr-1* may be chromosomally located or located on a non-mobile plasmid such as those described by Yang et al [30], Sellera et al [28] and Falgenhauer et al [60]. Further work is ongoing and will use whole genome sequencing and plasmid sequencing to determine the *mcr-1* location. Reports from other researchers have located the *mcr-1* to plasmids of varying types and sizes while others have reported a chromosomal location [18, 25, 26, 29] suggesting that *mcr-1* may be more ubiquitous than previously thought which could pose considerable concerns in how to control the spread of resistance and potential for treating disease in animals once the resistance becomes established in a host.

In conclusion, our report on the detection of *mcr-1* in APEC associated with colibacillosis in production poultry is relatively new, and we could find only three other studies by Perreten and colleagues [61] and Yang et al [30] reporting *mcr-1* in APEC in South Africa and China and a genome announcement of an avian ExPEC from Germany [31]. Regardless, our study would appear to be one of the first identifying *mcr-1* in APEC from China and Egypt of earlier dates than previously reported.

## Supporting information

S1 Fig Nucleotide alignment of the twelve strains positive for the *mcr-1* gene. (PDF)

S2 Fig Protein Alignment of *mcr-1* positive strains from examined in this study. (PDF)
